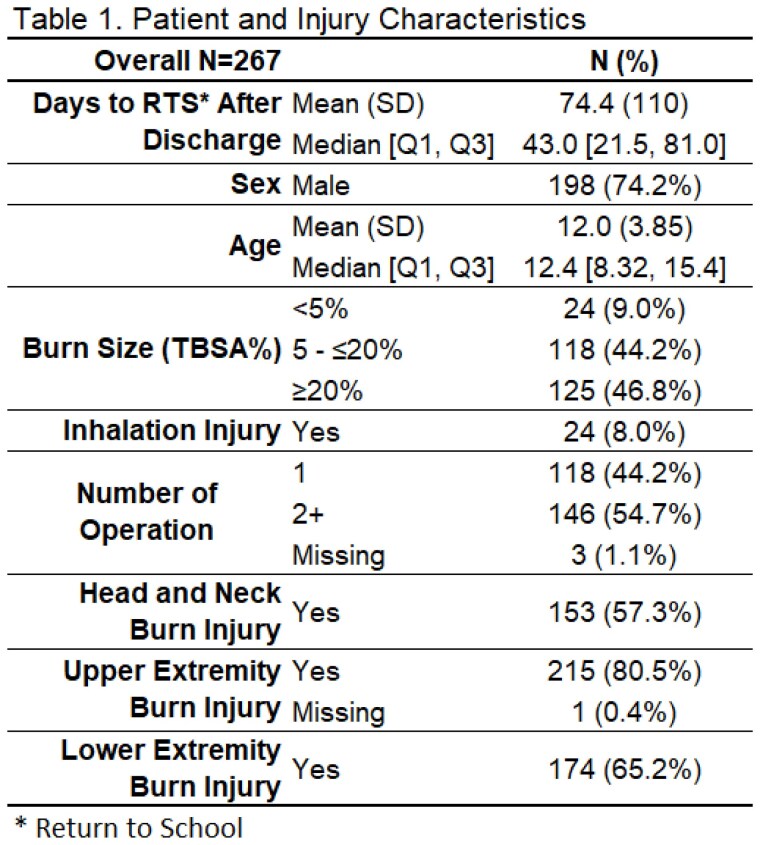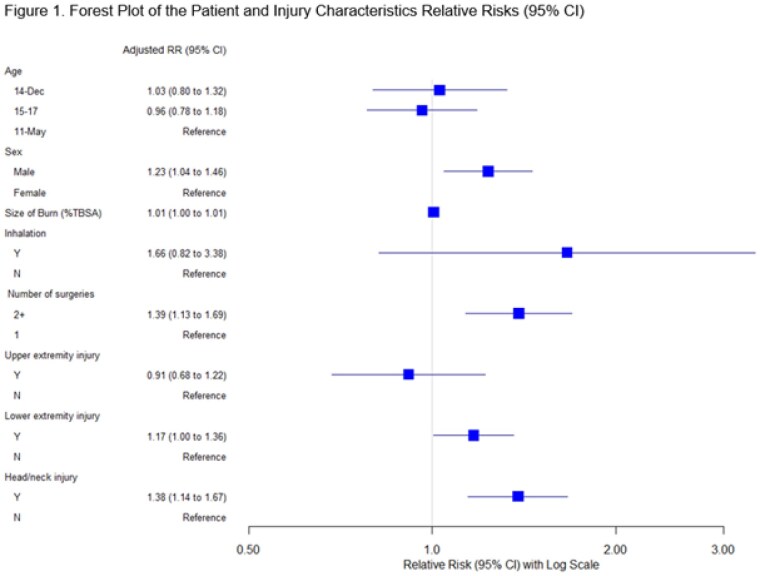# 73 Factors Impacting Delayed Return to School Among Children Living with Burn Injury: A Multicenter Analysis

**DOI:** 10.1093/jbcr/iraf019.073

**Published:** 2025-04-01

**Authors:** Geun-woo Oh, Gretchen Carrougher, Xinyao deGrauw, Deja Nicholas, Carly Marincasiu, Caitlin Orton, Shelley Wiechman, Sarah Stoycos, Colleen Ryan, Barclay Stewart

**Affiliations:** Harborview Medical Center, University of Washington; University of Washington; University of Washington, Wahington Department of Health; Harborview Medical Center, University of Washington; Harborview Medical Center, University of Washington; University of Washington; Harborview Medical Center, University of Washington; University of California Keck School of Medicine; Shriners Children’s - Boston and Massachusetts General Hospital; University of Washington

## Abstract

**Introduction:**

Return to school (RTS) is a fundamental indicator of rehabilitation effectiveness for school-age children with burn injury. Previous studies reported on the timing of RTS but have not clearly delineated how individual and injury characteristics impact RTS due to single-center and limited sample sizes. Our goal is to provide factors to consider when benchmarking days to RTS and allocating rehabilitation resources.

**Methods:**

We analyzed data from a multicenter database of US school-age children and their parents/guardians between ages 5-17 years who required surgery for wound closure. Days from index hospital discharge to RTS by self-/parent-guardian-report were recorded via surveys. The associations between days to RTS and age, sex, size of burn (%TBSA), inhalation injury, number of operations, and body region of injury were examined using multivariable mixed-effects Poisson regression analyses with robust standard error estimates.

**Results:**

This study included data from 267 participants (Table 1). The median days to RTS after hospital discharge was 74.4 (IQR, 21.5-81). Male sex (RR 1.23, 95%CI 1.04-1.46), head and neck burn (RR 1.38, 95%CI 1.14-1.67), lower extremity(ies) burn (RR 1.17; 95% CI, 1.0, 1.36), inhalation injury (RR, 1.66; 95% CI, 0.82, 3.38), and having 2+ operations for wound closure (RR 1.39, 95%CI, 1.13, 1.69) were significantly associated with longer time to RTS (Figure 1). Size of burn (%TBSA), age/grade, and upper extremity(ies) burn injury were not associated with longer RTS.

**Conclusions:**

Days to RTS after discharge in school-age children with burn injury were significantly longer among boys, children who required more operations, and injuries on head, neck, and lower extremities.

**Applicability of Research to Practice:**

Burn centers, schools, and burn camps may consider implementing and evaluating tailored support programs and accommodations, such as providing flexible school and exam schedules and burn injury care training for school nurses, that address child- and injury-specific risks for delayed RTS and needs for successful and timely school reintegration.

**Funding for the Study:**

The contents of this abstract were developed under a grant from the National Institute on Disability, Independent Living, and Rehabilitation Research (NIDILRR grant #90DPBU0005). NIDILRR is a Center within the Administration for Community Living (ACL), Department of Health and Human Services (HHS). The contents of this abstract do not necessarily represent the policy of NIDILRR, ACL, HHS, and do not assume endorsement by the Federal Government.